# Immune-Related Peripheral Neuropathy Associated with Immune Checkpoint Inhibitors: Case Report and Review of Literature

**DOI:** 10.1155/2024/8212943

**Published:** 2024-04-02

**Authors:** Carlos Eduardo Bonilla, Vaneza Ávila

**Affiliations:** ^1^Unidad Funcional de Tumores Gastrointestinales, Centro de Tratamiento e Investigación sobre Cáncer Luis Carlos Sarmiento Angulo (CTIC), Bogotá, Colombia; ^2^Grupo de Investigación GIGA, CTIC/Universidad El Bosque, Bogotá, Colombia; ^3^Unidad Funcional Asistencial Hospitalización, Centro de Tratamiento e Investigación sobre Cáncer Luis Carlos Sarmiento Angulo (CTIC), Bogotá, Colombia

## Abstract

Immune checkpoint inhibitors (ICIs) are a group of drugs that have improved outcomes for patients with various cancers. Generally considered safe and well tolerated, these drugs are occasionally linked to immune-mediated or immune-related adverse events. Among these, autoimmune neurological events are rare, displaying varying incidence rates across different studies. Peripheral neuropathy, although one of the more common neurological immune-related events, is at times underestimated. This case report highlights an adult patient diagnosed with metastatic intrahepatic cholangiocarcinoma. Initially, the patient underwent chemoimmunotherapy with gemcitabine, cisplatin, and durvalumab for eight cycles, achieving partial response without significant toxicity. Following this, the patient continued with maintenance monotherapy with durvalumab every 28 days. After completing six cycles of maintenance therapy, the patient suddenly experienced paresthesia and hypoesthesia in four limbs, accompanied by apraxia in the hands that was more pronounced on the right side. Additionally, the patient reported neuropathic pain in the right arm and encountered limitations in certain instrumental activities of daily living. Diagnostic studies, including laboratory and electrophysiological studies, combined with the clinical presentation, identified immune-related peripheral polyneuropathy. Durvalumab was suspended and prednisolone therapy was initiated, resulting in a rapid resolution of all neuropathic symptoms. In addition to the clinical case, this article reviews the literature on immunotherapy-associated peripheral neuropathy.

## 1. Introduction

Antitumor immunotherapy with immune checkpoint inhibitors (ICIs) represents a new cornerstone in oncology treatment, as it has demonstrated improvement in the outcomes in multiple cancers, including cholangiocarcinoma [1]. The mechanism of action of these drugs involves immune checkpoint blockade, enhancing antitumor immunity by inhibiting intrinsic suppressors of the immune system, such as cytotoxic T lymphocyte antigen 4 (CTLA-4), programmed cell death 1 (PD-1), or its ligand PD-L1 [2].

Generally, ICIs are considered safe and well tolerated; however, due to their mechanism of action, they are frequently associated with the occurrence of immune-related adverse events (irAEs), described in up to 90% of those receiving anti-CTLA-4 antibodies and 70% of those receiving anti-PD-1/PD-L1 [3].

Neurological immune-related adverse events (NirAEs) associated with immune checkpoint inhibitors (ICI) are rare, initially described in around 1% of patients receiving ICI. Still, the incidence varies across different series and has been increasingly reported in recent years. Among NirAEs, one of the most significant is immune-related peripheral neuropathy, which is sometimes underestimated [4].

Despite their low incidence, NirAEs, including immune-related peripheral neuropathy, require significant attention, as they can profoundly impact the quality of life and may even be fatal.

If a patient develops peripheral neuropathy symptoms during immunotherapy, immune-related peripheral neuropathy should be considered among the diagnostic possibilities.

## 2. Case Presentation

A 74-year-old man without previous comorbidities was diagnosed in September 2022 with metastatic intrahepatic cholangiocarcinoma featuring multiple focal hepatic lesions, extensive peritoneal carcinomatosis, and regional and extraregional lymph node involvement (retroperitoneal). Next-generation sequencing (NGS) of tissue revealed a stable microsatellite (MSS) tumor, a low mutational tumor burden, and a pathogenic mutation in dehydrogenase 1 (IDH-1) gene, R132C, 394C>T, with a variant allele frequency (%VAF) of 30%.

**Figure 1:**
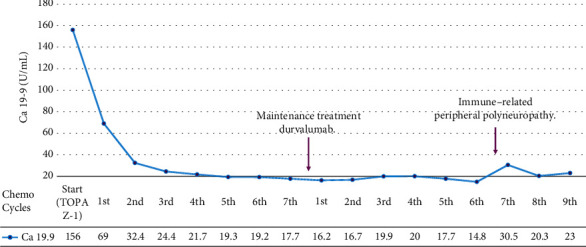
Serum cancer antigen CA 19.9 during treatment.

The patient initiated a first-line systemic treatment with cisplatin, gemcitabine, and durvalumab on September 26, 2022, achieving rapid symptom improvement, normalizing CA 19.9 after two cycles and sustained partial response on reevaluation images.  Figure 1 depicts the evolution of CA 19.9 antigen levels during treatment.

After completing eight cycles of chemoimmunotherapy, chemotherapy was discontinued, and maintenance immunotherapy with durvalumab was initiated on March 30, 2023.

In September 2023, during the sixth cycle of durvalumab monotherapy, the patient developed acute neurologic symptoms, including paresthesia, dysesthesia, and hypoesthesia in all four limbs. This was associated with weakness and apraxia in the hands, more pronounced on the right side, and neuropathic pain in the right upper limb, restricting some instrumental activities of daily living. Laboratory results revealed positive antinuclear antibodies (ANA) at a titer off 1/2560 with a granular fine pattern. Anti-DNA and the extractable antigen-antibody panel (Ro, La, Smith, RNP, and SCL-70) were negative. Other tests were within normal ranges, including CPK, thyroid function, parathyroid hormone, glycemia, vitamins B12 and D, and electrolytes. Electromyography and nerve conduction velocity test informed the following: absence of sensory response in the right median, radial, and sural nerves; reduced amplitude in the sensory nerve conduction of the left ulnar, median, and sural nerves, with prolonged latency in the right median nerve; motor nerve conduction in the median nerves with prolonged latency and reduced amplitude in the left motor potential; and motor nerve conduction in the left peroneal nerve with decreased amplitude and diminished conduction velocity. These findings were indicative of a mixed-pattern polyneuropathy predominantly of axonal type. Considering all clinical and paraclinical findings, a diagnosis of immune-related peripheral polyneuropathy associated with ICI was established and graded as two according to Common Terminology Criteria for Adverse Events (CTCAE) version 5.0.

Durvalumab was temporarily discontinued, and steroid therapy with prednisolone at a dose of 0.5 mg/kg/day was initiated, leading to symptom resolution within 72 hours. After a 20-day tapering period of the steroid dose, maintenance prednisolone therapy was set at 10 mg per day. With the resolution of symptoms, durvalumab was reintroduced in November 2023. As of the current date, the patient has received three more cycles of durvalumab without experiencing a recurrence of symptoms.

## 3. Discussion

Antitumor immunotherapy with ICI has been one of the most significant advances in oncology in recent years. Whether as a monotherapy or in combination with other treatment modalities, ICIs have improved outcomes in various cancers. This group of medications works by blocking receptors that inhibit the immune system (checkpoints), aiming to activate and boost an antitumor immune response [1].

Various immune checkpoint molecules have been identified, including the programmed cell death 1 receptor (PD-1), cytotoxic T lymphocyte antigen 4 (CTLA-4), PD-1 ligand (PD-L1), LAG3, TIGIT, TIM3, B7H3, CD39, CD73, or CD47. The first three are the ones that, up to this point, have the most advanced drug development [1].

In general, ICIs are considered safe and well-tolerated medications. However, due to their mechanism of action, they are frequently associated with the occurrence of immune-related adverse events (irAEs), which can vary in onset and severity. These events have been described in up to 90% of those receiving anti-CTLA-4 antibodies and 70% of those receiving anti-PD-1/PD-L1, and they are severe (*grade* ≥ 3) in approximately 20% of patients [2].

Neurological Immune-related adverse events (NirAEs) are considered infrequent and classically described in about 1% of patients [3]. However, they have been reported more frequently in recent years: 1 to 6% in those receiving monotherapy and up to 14% in those receiving ICI combinations [4]. Despite their low incidence, NirAEs require significant attention, as they can significantly impact the quality of life and may even be fatal [3].

A meta-analysis of 39 clinical trials found that ICI had a lower risk of neurological events compared to cytotoxic chemotherapy but a higher risk than the placebo. In this meta-analysis, the incidence of NirAEs ranged from 1 to 3.8%. However, they accounted for nearly 11% of fatal adverse events associated with ICIs [5].

NirAEs include manifestations that can affect the peripheral nervous system (PNS) or the central nervous system (CNS), with the former being more common. A recent consensus on NirAEs proposes classifying them as follows [6]:

(1) Central nervous system:
  - Aseptic meningitis
  - Encephalitis: meningoencephalitis, encephalomyelitis, cerebellitis, rhombencephalitis, etc.
  - Demyelinating syndromes: optic neuritis, transverse myelitis, acute disseminated encephalomyelitis, acute hemorrhagic encephalomyelitis
  - Vasculitis: primary CNS angiitis, systemic vasculitis with CNS involvement
(2) Peripheral nervous system:
  - Neuropathy: several subtypes or phenotypes, including Guillain-Barré syndrome and variants
  - Neuromuscular junction disorders: myasthenia gravis, Lambert-Eaton syndrome
  - Myopathy: immune-mediated necrotizing myopathy, inflammatory myopathy/myositis

The median number of ICI cycles in which NirAEs appear is 3; however, cases of late-onset after more than ten treatment cycles have been described. Possible risk factors for NirAEs include the use of anti-CTLA-4 antibodies, a history of autoimmune disease, and altered renal function. In contrast, female sex and the use of corticosteroids have been described as protective factors [4].

**Figure 2:**
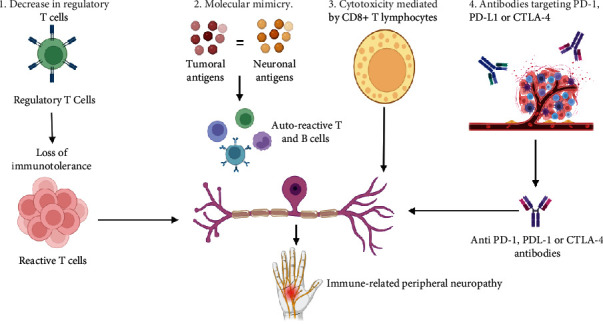
Possible pathophysiological mechanisms of immune-related peripheral neuropathy associated with ICI. Designed by the authors using the BioRender app (https://app.biorender.com/).

The heterogeneity in the presentation of NirAEs suggests that various pathophysiological mechanisms may be involved, including activation of effector T lymphocytes, a decrease in T-REG lymphocytes, molecular mimicry between normal and tumor cells, an excess of proinflammatory cytokines, dissemination of tumor epitopes, the eventual expression of PD-L1 and CTLA-4 in cells of the nervous system, some genetic polymorphisms in the immune system, and eventually some alterations in gastrointestinal microbiome. In Figure 2, the possible pathophysiological mechanisms of peripheral neuropathy associated with ICI are presented [7, 8].

Peripheral neuropathy is one of the most common neurological immune-related adverse events (NirAEs), accounting for almost half in some studies [4]. The spectrum of presentation is highly variable, with different symptoms and signs that may include dysesthesia, paresthesia, hypoesthesia, hyporeflexia or areflexia, altered proprioception, muscle weakness, neuropathic pain, allodynia, muscle cramps or fasciculations, involvement of cranial nerves, diplopia, dysphagia, dyspnea, changes in proprioception, imbalance, and ataxia [8–10].

According to neurological involvement, clinical manifestations, and the type of predominantly affected fiber, peripheral neuropathies can be classified into subtypes or phenotypes, among which are polyneuropathy, polyradiculopathy, axonal polyradiculoneuropathy (radiculoplexus neuropathy), acute inflammatory demyelinating neuropathy (Guillain-Barré syndrome and variants), chronic inflammatory demyelinating neuropathy, cranial neuropathy, small fiber/autonomic neuropathies, sensory neuronopathy, plexopathies, mononeuritis multiplex, neuralgic amyotrophy, and acute length-dependent sensorimotor polyneuropathy [6, 11, 12].

The diagnostic approach to immune-related neuropathies typically requires clinical correlation with laboratory tests, imaging, electrodiagnostic studies, and, in some cases, biopsies [6].

Based on symptom evaluation and physical examination, considering the potential phenotype, the following tests can be used for diagnostic support and to rule out alternative causes [6, 10].

Within the laboratory tests, some of the following can be considered: HbA1c, blood glucose, vitamin B12, vitamin B6, thiamine, folates, thyroid function tests, serum protein electrophoresis and immunofixation, total CPK, antinuclear antibodies, extractable antibodies (Ro, La, Sm, and RNP), anti-DNA, ANCA, anti-MAG, anti-Hu, anti-ganglioside antibodies, electrolytes, syphilis treponemal tests, studies for HIV, and hepatitis B and C. Sometimes, images are needed, such as magnetic resonance imaging (MRI) of the brain, spine, cranial plexuses, or peripheral nerves.

A lumbar puncture can be helpful in some cases, as it allows for the exclusion of tumoral or infectious causes. In cases of immune-related neuropathy, lymphocytic pleocytosis and elevated protein levels in the cerebrospinal fluid (CSF) are common; however, these findings are nonspecific.

Electrodiagnostic testing with electromyography and nerve conduction velocities is crucial in evaluating a potential immune-related peripheral neuropathy. These tests allow for assessing the presence of large fiber neuropathies; defining the type, severity, and chronicity of the neuropathy; and ruling out other possible causes of the symptoms [6]. The most common finding is a pattern of demyelination with decreased conduction velocities, temporal dispersion (sometimes conduction block), prolonged motor distal latency, and prolonged F-wave latencies; however, an axonal or mixed pattern may also be found in some cases [7, 12].

Immune-related peripheral neuropathy is usually asymmetric, with acute or subacute onset, exhibits a length-independent pattern, and often shows some degree of motor involvement from the beginning [12], in contrast to chemotherapy-induced neuropathy, which tends to be more symmetric and insidious and more frequent of the axonal type [6, 12].

A consensus on NirAEs [6] considers that for a definitive diagnosis of immune-related peripheral neuropathy, the following three criteria are required:

1. Abnormal electrodiagnostic studies demonstrating a neuropathy subtype associated with ICI
2. Laboratory or imaging evaluation showing evidence of an immune-mediated cause for the neuropathy
3. Absence of alternative causes that better explain neuropathy

Other studies such as autonomic function tests or nerve biopsy are not indispensable, but they can be supportive if done.

In cases where the clinical presentation and electrodiagnostic findings are suspicious, but there is another etiology that could better explain the findings, the diagnosis is considered probable. If there are clinical criteria without supporting laboratory or electrodiagnostic data, the diagnosis is only regarded as possible [6].

The management of autoimmune peripheral neuropathy primarily depends on the severity. Severity grading of irAEs is mainly done using the Common Terminology Criteria for Adverse Events (CTCAE) scale. Although developed during the era of other oncologic treatments, this scale can also be applied to events associated with immunotherapy, including those affecting the nervous system [13].

For sensory or motor peripheral neuropathy, the severity is classified as grade 1 (asymptomatic), grade 2 (moderate symptoms, with limitation for instrumental activities of daily living), grade 3 (severe symptoms, with limitations in self-care activities), grade 4 (life-threatening consequences), and grade 5 (death).

The main guidelines for the management of immune-related events associated with ICI [10, 14] consider the following management for patients with immune-related peripheral neuropathy:

In grade 1, ICI may be continued with close monitoring, without additional treatment.

For grade 2 neuropathy, it is recommended to withhold ICI and initiate corticosteroid management with prednisolone at 0.5 to 1 mg/kg/day or its equivalent. There is a possibility of restarting ICI when the severity is grade 1 or lower.

For severe neuropathy (grades 3 or 4), it is recommended to permanently discontinue the ICI, hospitalize, and initiate treatment with methylprednisolone. If rapid improvement is not achieved or there are signs of life-threatening conditions, additional therapies such as plasma exchange, immunoglobulins, and rituximab, among others, may be considered [10, 14]. Considering the prolonged half-life of ICIs, it is recommended to maintain steroids for at least 6 to 8 weeks [10, 15].

**Figure 3:**
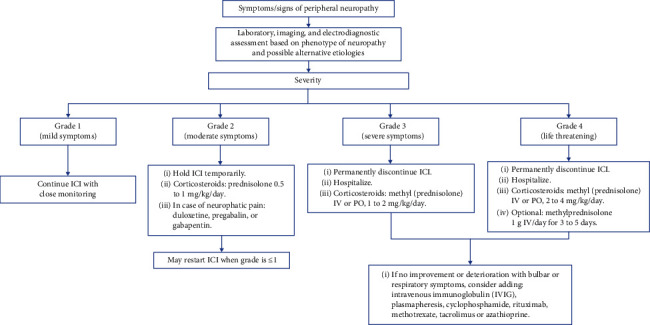
Algorithm for the initial treatment of immune-related peripheral neuropathy associated with ICI.

In Figure 3, a management algorithm for immune-related peripheral neuropathy is presented, adapted from the main guidelines [10, 14].

Most patients will experience rapid improvement with the use of corticosteroids, but fatal events have been reported in about 20% of patients with severe (grades 3-4) NirAEs [16]. Early diagnosis and timely treatment initiation are essential for these patients.

In conclusion, we present the case of a man with metastatic cholangiocarcinoma who developed immune-related peripheral neuropathy during maintenance therapy with durvalumab. This condition should be considered in the differential diagnosis when a patient receiving ICI presents new neuropathic symptoms.
